# Clinical isolates from chronic wounds reveal strain-specific, alkyl-quinolone-independent competition in Pseudomonas aeruginosa–Staphylococcus aureus biofilms

**DOI:** 10.1099/jmm.0.002118

**Published:** 2026-01-14

**Authors:** Bethan Roberts, Ana C. da Silva, Tim Sloan, Christopher N. Penfold, Paul Williams, Stephen P. Diggle, Kim R. Hardie

**Affiliations:** 1 Biodiscovery Institute, School of Life Sciences, University of Nottingham, Nottingham, UK; 2 Centre for Microbial Dynamics and Infection, School of Biological Sciences, Georgia Institute of Technology, Atlanta, GA, USA; 3 Department of Biochemistry, University of Cambridge, Cambridge, UK; 4 Department of Clinical Microbiology, Nottingham University Hospital, Nottingham, UK

**Keywords:** biofilm, chronic wound, clinical isolates, 2-alkyl-4-quinolone molecules, *P. aeruginosa*, *S. aureus*

## Abstract

**Introduction.** Chronic wounds are notoriously difficult to treat and are associated with decreased limb function, reduced quality of life and significant morbidity. Their recurrent nature, despite aggressive antibiotic therapy, is due in part to the presence of polymicrobial biofilms. *Pseudomonas aeruginosa* and *Staphylococcus aureus* are two of the most frequently co-isolated pathogens in these infections and are known to form complex biofilms that hinder treatment.

**Hypothesis.** We hypothesized that co-existence and competitive dynamics between *P. aeruginosa* and *S. aureus* in chronic wound infections are influenced by strain-specific interactions and may not rely solely on well-characterized inhibitory mechanisms such as 2-alkyl-4-quinolone (AQ) production by *P. aeruginosa* impacting on *S. aureus* fitness.

**Aim.** To establish a polymicrobial chronic wound infection model and assess the contribution of AQ signalling and strain-specific interactions on co-existence.

**Methodology.** We used a modified chronic wound biofilm model to co-culture matched and mismatched clinical isolate pairs of *P. aeruginosa* and *S. aureus*, collected from two different chronic wound patients. Viable bacterial counts (c.f.u.) were quantified over an 8-day period. AQ production by each *P. aeruginosa* strain was quantified using liquid chromatography-MS.

**Results.** A stable culture of *P. aeruginosa* strains was achieved, but distinct behaviours between each *S. aureus* strain were seen. One matched clinical isolate pair maintained stable c.f.u. levels of both species throughout the 8-day model, indicating a compatible co-existence. In contrast, mismatched pairs showed early loss of *S. aureus* viability and the emergence of small colony variants after 4 days, not seen in matched pair growth. Interestingly, the most competitive *P. aeruginosa* strain exhibited undetectable levels of all AQs tested, indicating that its dominance was not due to AQ-mediated antagonism, as has previously been described.

**Conclusion.** Our findings demonstrate that stable dual-species biofilm formation in chronic wounds is strain-dependent and that *P. aeruginosa* can impact on *S. aureus* fitness through AQ-independent mechanisms. These results highlight the importance of using clinical isolates in biofilm research and caution against generalizing findings from laboratory strains to complex clinical infections.

## Introduction

*Pseudomonas aeruginosa* and *Staphylococcus aureus* are frequently co-isolated from chronic wounds, the sputum of individuals with cystic fibrosis (CF) and various other infection sites in both humans and animals. Bacterial prevalence in CF lungs shows *S. aureus* predominates in childhood, whereas *P. aeruginosa* becomes the dominant pathogen in young adulthood, although *S. aureus* frequently persists at significant levels [1,2]. Co-isolation of both species is common in adults with CF. In chronic wound infections, *P. aeruginosa* generally colonizes the deeper layers of the wound bed, while * S. aureus* is more commonly found at the wound surface [3]. Despite occupying distinct spatial niches, interactions between the two species have been previously suggested [4]. Historical studies have emphasized the competitive dominance of * P. aeruginosa* over *S. aureus* [5,7], but more recent clinical evidence indicates that these two species can co-exist within chronic wounds and may influence disease outcomes [8,10]. Within polymicrobial biofilms, *P. aeruginosa* and *S. aureus* can benefit from each other through the secretion of signalling molecules and the shared competition for space and nutrients. Such interactions may enhance host colonization and modulate immune responses in ways that affect wound healing and increase the overall virulence of both species [11].

Previous studies using laboratory strains of *P. aeruginosa* and *S. aureus* have revealed several mechanisms of interspecies antagonism during co-culture. *P. aeruginosa* secretes small-molecule exoproducts, such as 2-alkyl-4-quinolones (AQs) and *N*-acylhomoserine lactones, which co-ordinate quorum sensing (QS). This regulatory network controls the expression of virulence genes, the production of secondary metabolites and biofilm formation [12]. As a result, *P. aeruginosa* can influence the survival and susceptibility of other bacterial species in dual-species infections. Antagonism towards *S. aureus* by * P. aeruginosa* has been linked to several factors, including AQs such as 2-heptyl-4-hydroxyquinoline-*N*-oxide (HQNO), 2-heptyl-3-hydroxy-4(1*H*)-quinolone (Pseudomonas quinolone signal, PQS) and 2-heptyl-4-hydroxyquinoline (HHQ), as well as phenazines, pyocyanin and hydrogen cyanide. These compounds can disrupt *S. aureus* respiration and viability [10,13]. In response, *S. aureus* can adapt by forming small colony variants (SCVs), which are characterized by slow growth, reduced metabolic activity and decreased membrane potential. These features confer resistance to antibiotics, host antimicrobial peptides and toxic bacterial metabolites [14]. SCVs are capable of persisting in polymicrobial environments and can continue to modulate the behaviour and gene expression of neighbouring species. Despite this progress, the precise mechanisms underlying *P. aeruginosa*–*S. aureus* interactions *in vivo* remain poorly understood, largely due to a lack of studies using clinical strains isolated from the same or different patients.

*In vitro* co-culture experiments involving *P. aeruginosa* and *S. aureus* have revealed that the spatial distribution of these species varies depending on their molecular interactions [3]. Segregation is often observed, with *P. aeruginosa* forming biofilms at the air–liquid interface, while *S. aureus* tends to settle at the bottom of the well [15]. In contrast, analyses of chronic wound biopsies have shown that *P. aeruginosa* aggregates are typically located deeper within the wound bed, whereas *S. aureus* is found closer to the surface [16]. Clinical isolates have also demonstrated co-localization of the two species *in vitro*, which is thought to arise as a stress response to antibiotic treatment or host immune pressure [17]. However, the concept of co-localization remains controversial, with some studies reporting the presence of mono-species biofilm aggregates even in polymicrobial infections [18]. These conflicting findings underscore the influence of culture conditions on bacterial organization and highlight the challenges of translating *in vitro* observations to *in vivo* infections. As a result, our understanding of dual-species bacterial interactions in clinically relevant settings remains limited. To bridge this gap, experimental models should increasingly incorporate clinical isolates directly obtained from infected sites.

In this study, we investigated the interaction between *P. aeruginosa* and *S. aureus* strains using two pairs of clinical isolates obtained from human chronic wound ulcers. Matched and mismatched isolate pairs were co-cultured in a modified chronic wound biofilm (mCWB) model, revealing distinct *S. aureus* behaviours [19]. Stable co-culture was observed for one of the matched clinical pairs, acknowledging the mCWB model as a realistic *in vitro* system for studying chronic wound infections. In contrast, when this pair was mismatched, a stable dual-species biofilm was unattainable, with rapid decline of *S. aureus*, suggesting strain-specific incompatibilities that influence survival in chronic infection contexts. Notably, quantification of AQ production revealed that the most competitive *P. aeruginosa* strain produced negligible AQ levels. This finding suggests that the observed dominance in co-culture may not be driven by AQ-mediated mechanisms but rather by alternative, yet-to-be-identified competitive strategies.

## Methods

### Bacterial strains and growth conditions

The strains used in this study are detailed in Table 1. Unless stated otherwise, growth of all bacterial species was in peptone water to more realistically mimic the chronic wound environment. A list of the media used in this study is detailed in Table 2. Bacteria were routinely grown at 37 °C, 200 r.p.m. in peptone water for liquid culture or at 37 °C statically for growth on lysogeny broth (LB) agar plates. Bacterial growth in liquid media was assessed using OD at 600 nm (OD_600nm_).

**Table 1.** List of strains used in this study

Organism	Description	Source
*P. aeruginosa* CW2 (Bone)	Isolated from a chronic wound infection via bone biopsy alongside S.A. CW2. Resistance to amoxicillin, co-amoxiclav and ceftriaxone. Intermediate resistance to aztreonam.	Patient V, Nottingham University Hospitals
*P. aeruginosa* CW4 (Tissue)	Isolated from a chronic wound infection via tissue biopsy alongside S.A. CW4. Resistant to ciprofloxacin, erythromycin, amoxicillin and flucloxacillin. Intermediate resistance to aztreonam.	Patient X, Nottingham University Hospitals
*S. aureus* CW2	Isolated from a chronic infection alongside P.A. CW2. Resistant to doxycycline, methicillin sensitive.	Patient V, Nottingham University Hospitals
*S. aureus* CW4	Isolated from a chronic infection alongside P.A. CW4. Methicillin-resistant.	Patient X, Nottingham University Hospitals
*P. aeruginosa* PAO1L	*P. aeruginosa* PAO1 Lausanne sub-strain.	[34]

**Table 2.** List of media used in this study

Media	Use	Preparation
**Peptone water**	Overnight growth of strains for mCWB model	Made according to manufacturer’s instructions (Oxoid), then autoclaved in-house by technicians
**PIA**	Isolation of *P. aeruginosa* from a mixed culture	Made according to manufacturer’s instructions (Merck Life Science UK), then autoclaved in-house by technicians
**MSA**	Isolation of *S. aureus* from a mixed culture	Made according to manufacturer’s instructions (Oxoid), then autoclaved in-house by technicians
**WSM**	For use in the mCWB model	50% (v/v) foetal bovine serum (Gibco), 50% (v/v) peptone water at 0.1% (w/v)

### The mCWB model

The mCWB model used in this study was developed by Thaarup *et al.* [19] and consists of a dermis-like layer on top of a subcutaneous fat-like layer, and the two bacterial species are inoculated on top of the dermis layer on the same day. The semi-solid media were cast in a 12 mm transwell with a 0.4 µm pore (Corning) allowing continual nutrient supplementation over the course of the experiment. Nutrients are provided by wound simulating media (WSM) in the lower chamber of the transwell. The model was set up as described by Thaarup *et al.* [19]. However, overnight cultures of the appropriate bacterial species for this study were grown in peptone water rather than LB as stated in [19], which was prepared according to the manufacturer’s instructions and autoclaved (Oxoid).

### Homogenization of mCWB media and c.f.u. determination

C.f.u.s were determined by homogenizing the mCWB semi-solid medium after removal from each transwell using a pipette and adding it to a 2 ml reinforced tube (Fisherbrand) containing six ceramic beads (2.8 mm). A volume of 1 ml PBS was added to each tube and the samples were beaten for 2×10 s at 6,000 r.p.m. using a Bead Mill 24 (Fisherbrand). The medium was transferred to a 2 ml tube, and 300 µl collagenase (500 mg ml^−1^; Merck) was added. The tubes were briefly vortexed and incubated at 37 °C for 1 h to allow the collagenase to digest the collagen matrix. For c.f.u.s, 100 µl of each sample was serially diluted in 900 µl PBS, and 10 µl samples were plated, in triplicate, onto both Pseudomonas isolation agar (PIA) (Millipore), which selects for *P. aeruginosa*, and Mannitol salt agar (MSA) (Oxoid), which selects for *S. aureus*. PIA plates were incubated at 22 °C overnight to minimize overgrowth of the *P. aeruginosa* c.f.u.s, and MSA plates at 37 °C until colonies were visible to count.

### Quantification of AQ molecules by liquid chromatography-MS

The production of six AQ molecules [HHQ, 2-nonyl-4-hydroxyquinoline (NHQ), HQNO, 2-nonyl-4-hydroxyquinoline-*N*-oxide (NQNO), PQS and 2-nonyl-3-hydroxy-4(1*H*)-quinolone (C9-PQS)] was quantified. P.A. CW2 and P.A. CW4 were grown in LB overnight then seeded at OD_600nm_ 0.01 in 10 ml of WSM and incubated at 37 °C, 200 r.p.m. for 18 h. Samples were centrifuged at 4,000 r.p.m. for 10 min and filtered using a 2 ml syringe with a 0.2 µm filter (Minisart). Ten microlitres of 10 µM 5,6,7,8-tetradeutero-3,4-dihydroxy-2-heptylquinoline was used as an internal standard and was added in 500 µl of acidified (0.1% acetic acid) ethyl acetate (Sigma Aldrich, UK) to 500 µl of the filtered supernatant. The mixture was vortexed for 2 min and the organic phase removed. The extraction process was repeated twice, and the ethyl acetate extracts were evaporated in a speed vacuum (Jouan RC10 series, Thermo Scientific, UK) for 20 min at 45 °C. Samples were reconstituted in 50 µl methanol, and liquid chromatography-MS (LC-MS/MS) analysis was conducted according to [20]. PAO1L grown in WSM was used as a control.

### Statistics

Data were collected and analysed using Microsoft Excel (2018 or later) and GraphPad Prism 9. GraphPad was used to conduct statistical analysis and create graphs.

## Results

### Isolation of clinical strains from chronic wound infections

Two *P. aeruginosa* and two *S. aureus* strains were isolated from patients with chronic diabetic foot ulcers representing either short- or long-term infections, defined by the presence of *P. aeruginosa* for less than or more than 6 months, respectively. The matched clinical isolate pair *P. aeruginosa* (P.A. CW2) and *S. aureus* (S.A. CW2) were recovered from Patient V, while *P. aeruginosa* (P.A. CW4) and *S. aureus* (S.A. CW4) were isolated from Patient X (Table 1). P.A. CW2 and S.A. CW2 were obtained via the same bone biopsy from a big toe ulcer in a 78-year-old patient (Patient V) who also presented with osteomyelitis. The *P. aeruginosa* infection had persisted for 3 months and was therefore classified as short-term (Table 3). Despite medical intervention, the patient required toe amputation due to disease progression. Clinical swabs confirmed the continued presence of both *P. aeruginosa* and *S. aureus* in the wound for 2 months prior to surgery and strain isolation. P.A. CW4 and S.A. CW4 were isolated from a tissue biopsy of a calcaneal ulcer in Patient X, who had been infected with *P. aeruginosa* for 25 months, a long-term infection. This patient also had osteomyelitis, and the strain subsequently caused bloodstream infection (bacteraemia). Treatment was complicated by antimicrobial resistance and drug allergies, ultimately resulting in a below-knee amputation. There is clear evidence from clinical swabs of co-localization of P.A. CW4 and S.A. CW4 at least 6 months prior to isolation. To evaluate whether co-existence with a specific *S. aureus* strain affects bacterial survival *in vitro*, matched and mismatched isolate pairs were tested in the mCWB model.

**Table 3.** Patient infection information for each clinical isolate strain. OM: osteomyelitis at time of sampling. Duration: time since first recorded identification of *P. aeruginosa* or *S. aureus* in the wound. LT: long-term infection defined by the presence of *P. aeruginosa* for more than 6 months

Patient	Strains	Wound location	OM	Duration (months)	LT
**V**	*P. aeruginosa* CW2 *S. aureus* CW2	Big toe	Yes	3	No
**X**	*P. aeruginosa* CW4 *S. aureus* CW4	Calcaneal ulcer	Yes	25	Yes

### *In vivo* co-existence in the mCWB model affects bacterial survival

**Fig. 1.**
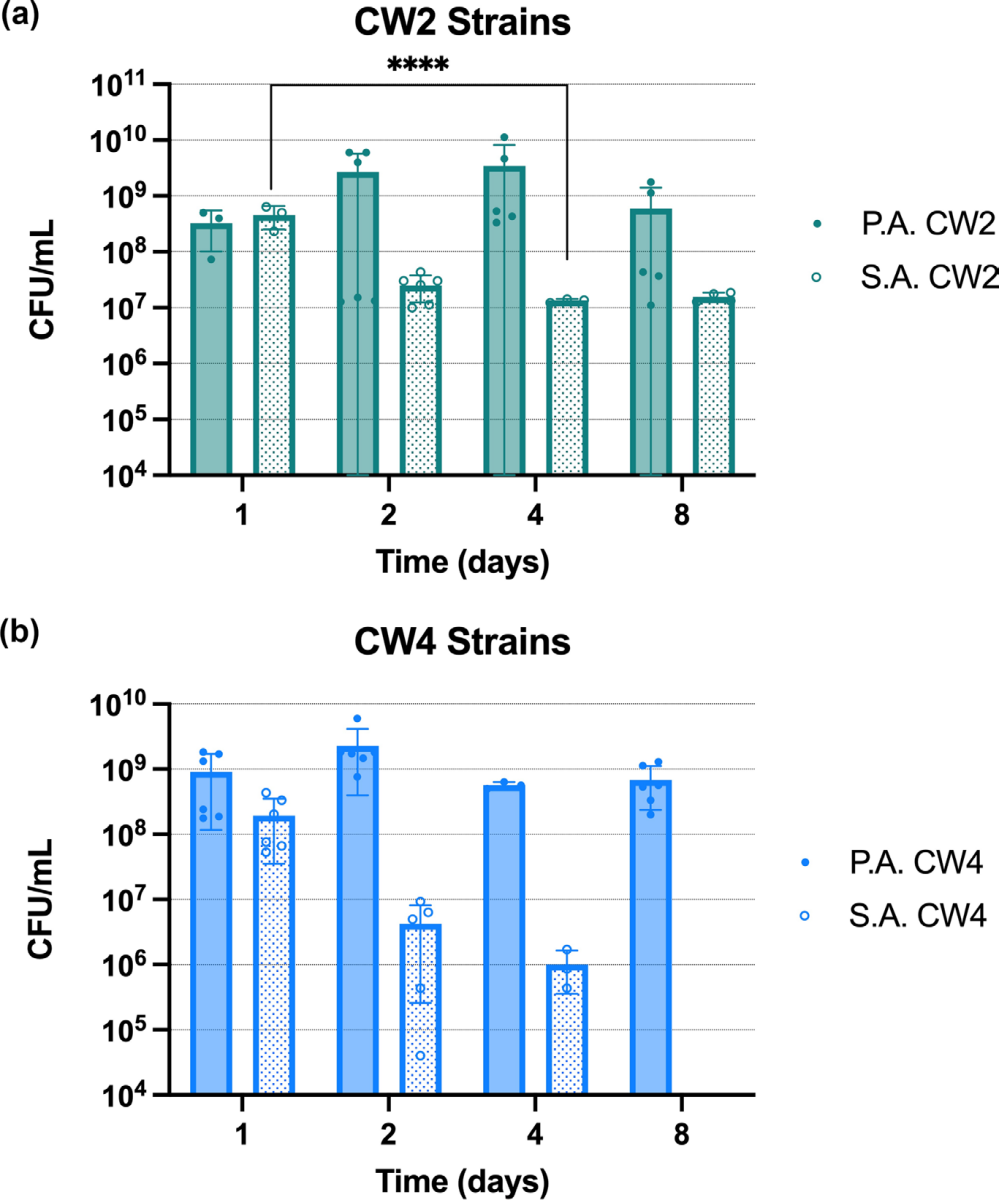
Matched clinical pairs grown in the mCWB model showed *S. aureus* strain-specific trends. Pairs of *P. aeruginosa* and *S. aureus* strains were co-inoculated into the mCWB model, and c.f.u.s were enumerated after 1, 2, 4 or 8 days incubation. The pairs constituted clinical isolate matched pairs that were recovered from the same patient: P.A. CW2 with S.A. CW2 (Panel A) and P.A. CW4 with S.A. CW4 (Panel B). The c.f.u.s for each strain are shown separately. A mixed-effects analysis with Dunnett’s multiple comparisons was conducted for each strain using day 1 c.f.u.s as control. ***P<0.001. *n*=6 biological repeats, mean±sd bars*.*

**Fig. 2.**
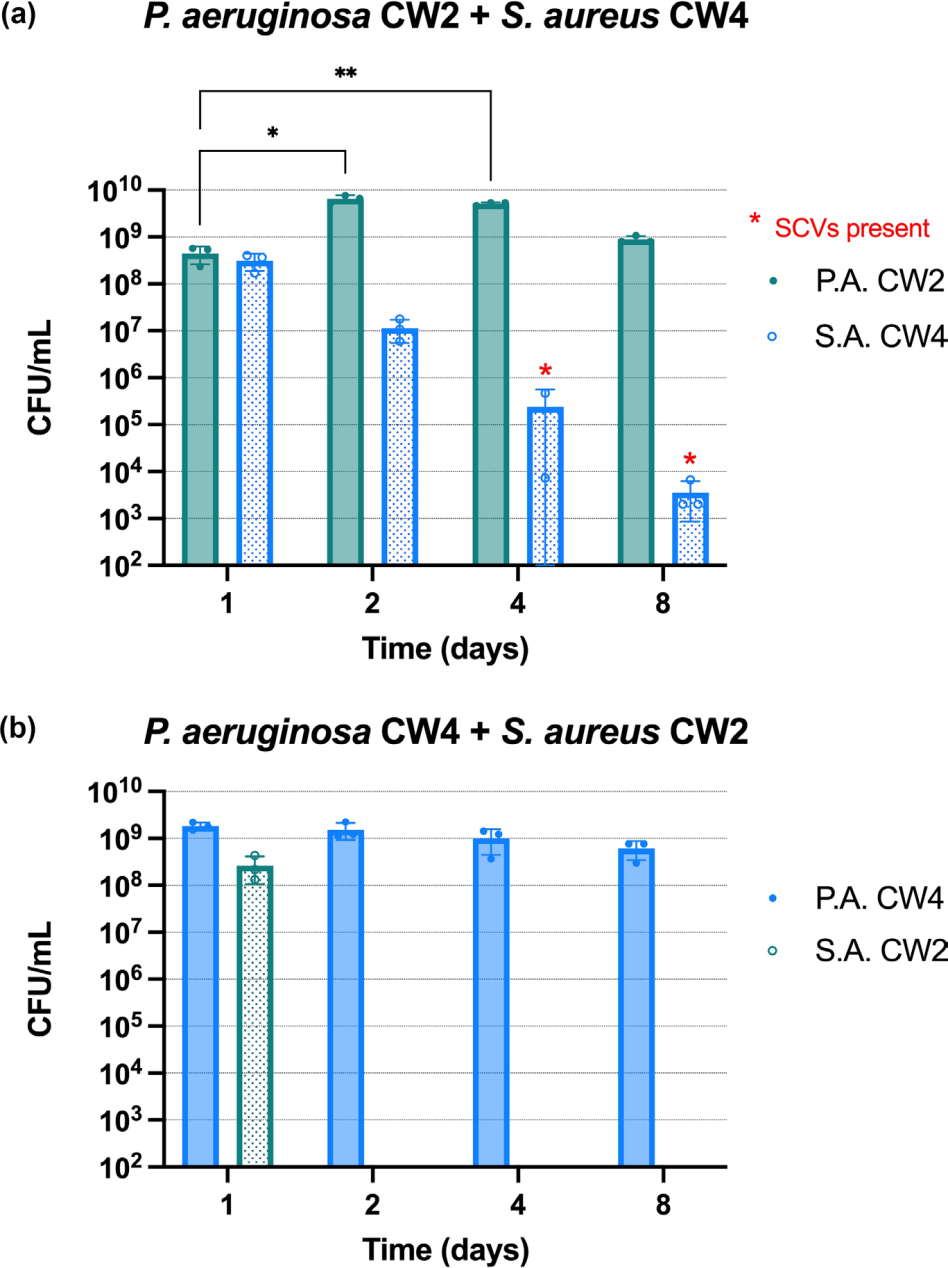
Mismatched clinical isolate pairs are unable to develop stable dual-species biofilms in the mCWB model. Pairs of *P. aeruginosa* and *S. aureus* strains were co-inoculated into the mCWB model, and c.f.u.s were enumerated after 1, 2, 4 or 8 days incubation. The pairs constituted clinical isolate mismatched pairs that were recovered from different patients: P.A. CW2 with S.A. CW4 (Panel A) and P.A. CW4 with S.A. CW2 (Panel B). The c.f.u.s for each strain are shown separately. SCVs were seen on days 4 and 8 by S.A. CW4, designated by a red asterisk. C.f.u.s were below the detection limit from day 2 for either S.A. CW2 strains. A mixed-effects analysis with Dunnett’s multiple comparisons was conducted for each strain using day 1 c.f.u.s as control. *P<0.05, **P<0.01. *n*=3 biological repeats, mean±sd bars*.*

Matched isolate pairs were initially tested to establish baseline viability in the mCWB model. P.A. CW2 was co-cultured with S.A. CW2 and P.A. CW4 with S.A. CW4. As shown in Fig. 1, both *P. aeruginosa* strains maintained stable c.f.u.s over the 8 day experiment with no significant changes in cell number seen over the course of the experiment. The two *S. aureus* strains, however, exhibited distinct behaviours. S.A. CW2 had a drop in c.f.u.s, which became significant on day 4 when compared to day 1, but maintained this stable drop from day 2 onwards, consistent with stable co-existence (Fig. 1a). In contrast, S.A. CW4 displayed a progressive decline in c.f.u.s, becoming undetectable by day 8, despite stable c.f.u.s of P.A. CW4 throughout the experiment (Fig. 1b). To further assess strain compatibility, mismatched pairs were also tested. When P.A. CW2 was co-cultured with S.A. CW4, the latter declined steadily and became barely detectable by day 8, whereas P.A. CW2 levels significantly increased on days 2 and 4 compared to day 1 (Fig. 2a). SCVs of *S. aureus* were observed at days 4 and 8, reverting to wild-type morphology after two passages without *P. aeruginosa* on LB agar. In the reciprocal mismatch, P.A. CW4 with S.A. CW2, *S. aureus* c.f.u.s dropped below detection levels by day 2, despite similar initial counts on day 1 (Fig. 2b). In comparison, the matched CW2 pair showed stable c.f.u.s for both species over time (Fig. 1). Notably, S.A. CW4 showed a slower decline and no SCV formation when paired with its matched P.A. CW4 strain.

### Competitive dominance of P.A. CW4 is independent of alkyl-quinolone production

**Fig. 3.**
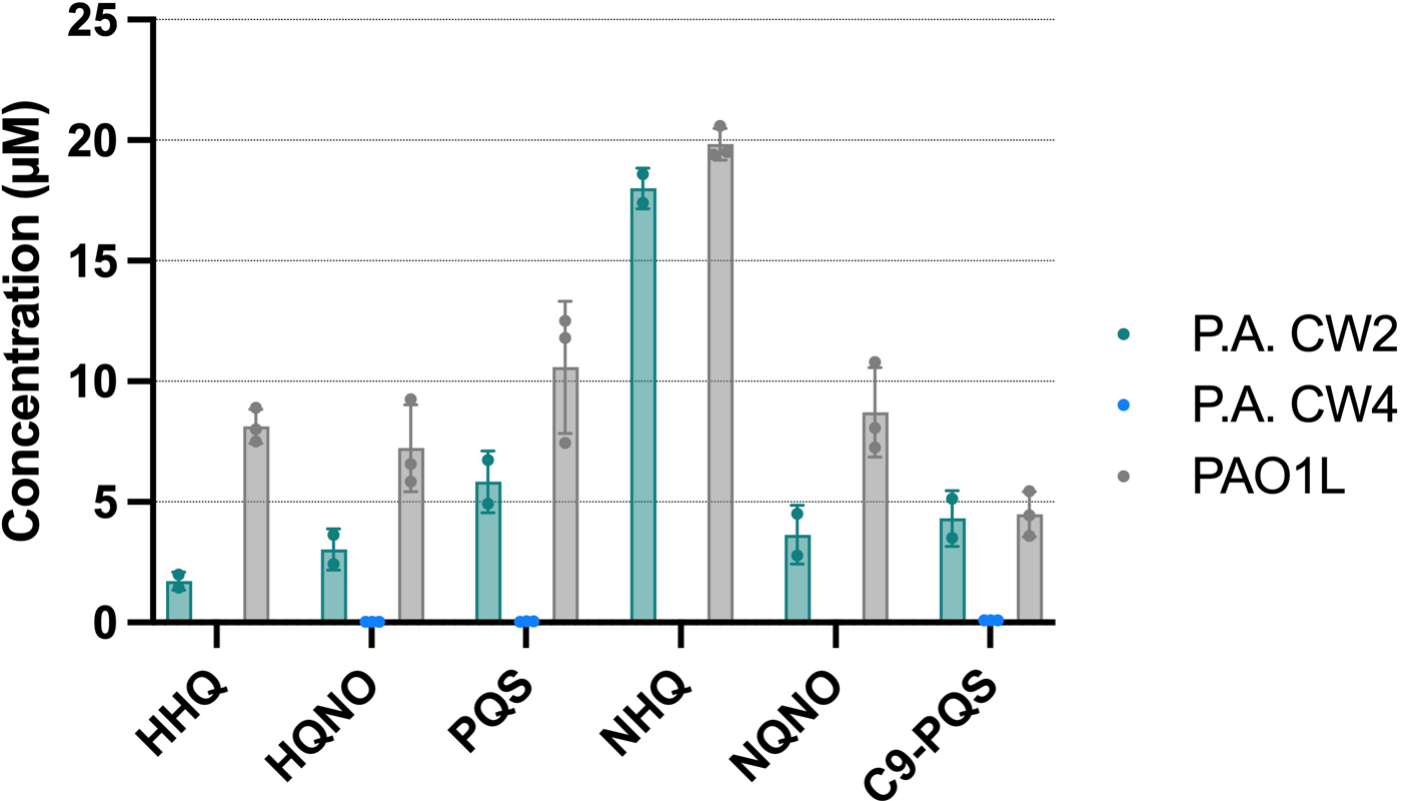
LC-MS/MS quantification of AQs produced by *P. aeruginosa* strains. *P. aeruginosa* P.A. CW2, P.A. CW4 and PAO1L were grown planktonically in WSM for 16 h, then levels of AQ molecules were quantified. Limit of detection was 0.5 µM. *n*=3 biological repeats for P.A. CW4 and PAO1L, *n*=2 for P.A. CW2, mean±sd bars*.*

Previous studies using the laboratory strain *P. aeruginosa* PAO1 have shown that AQs, including PQS, HHQ and HQNO, contribute to the killing of *S. aureus* during co-culture through disruption of respiration and metabolic activity. These QS-regulated secondary metabolites are considered key mediators of *P. aeruginosa* antagonism in polymicrobial infections. To determine whether a similar mechanism underlies the observed killing of S.A. CW2 by P.A. CW4, we quantified AQ production by each clinical isolate using LC-MS/MS. After overnight growth in WSM, P.A. CW2 produced detectable levels of five out of six AQs (HHQ, HQNO, PQS, NHQ, NQNO and C9-PQS), with all except HHQ comparable to the PAO1L control (Fig. 3). Surprisingly, P.A. CW4 produced undetectable levels of all six AQs tested (Fig. 3). For each AQ, levels were below 0.5 µM – the lowest calibration point and considered the minimum threshold for biological activity [21,22]. This finding suggests that P.A. CW4’s competitive advantage in co-culture is not due to classical AQ-mediated mechanisms but may involve alternative, as yet unidentified pathways.

## Discussion

The isolation of two paired clinical isolates of *P. aeruginosa* and *S. aureus*, each derived from separate chronic wound infections, provided us with a unique opportunity to investigate interspecies interactions using both matched (from the same patient biopsy) and mismatched (from different patients) combinations. This experimental design enabled not only the exploration of strain-specific compatibility but also the validation of the mCWB model using clinically relevant isolates, an approach rarely addressed in prior studies. By exchanging the *S. aureus* and *P. aeruginosa* isolates between pairs, mismatched co-cultures were generated to assess whether co-existence influences bacterial survival and to determine the potential role of AQ-mediated killing. Because the clinical isolates originated from infections of differing durations (short-term and long-term), this approach also allowed investigation into how prolonged co-culture might influence susceptibility to antagonism. Marked differences in bacterial survival were observed between the *S. aureus* strains when grown in mismatched pairings. S.A. CW2, isolated from a short-term infection, failed to persist in the presence of P.A. CW4, a strain from a long-term infection. In contrast, S.A. CW4 maintained viability when co-cultured with P.A. CW2, despite a reduction in c.f.u.s over the course of the experiment. These outcomes suggest that infection history and strain adaptation play critical roles in determining competitive outcomes and that stable dual-species biofilm formation in the mCWB model is strain-dependent.

Surprisingly, despite its greater competitiveness, P.A. CW4 produced negligible quantities of AQs, including HHQ, PQS, HQNO, NHQ, NQNO and C9-PQS. In contrast, P.A. CW2 produced measurable quantities of all but one AQ, with levels comparable to the PAO1L laboratory control. Genomic analysis of P.A. CW4 revealed multiple inactivating mutations in the PQS biosynthesis pathway, the *psqR* response regulator gene, as well as mutations in *phnA*, which is involved in anthranilate biosynthesis [23]. This finding is consistent with loss of AQ production in this strain. Based on previous literature where AQ molecules, particularly HQNO, have been implicated in *S. aureus* killing via respiratory inhibition, it was hypothesized that S.A. CW2 would be more resistant to P.A. CW4 due to the latter’s reduced AQ profile [24]. However, this was not the case, indicating that P.A. CW4 employs alternative, AQ-independent killing mechanisms. This observation suggests that other virulence factors may contribute to * P. aeruginosa*-mediated antagonism. For example, polyphosphate (polyP), a biopolymer targeting the *S. aureus* cell envelope, and LasA protease, which causes lysis of Gram-positive bacteria, are known to play roles in interspecies competition [25,26]. The expression or activity of such factors may explain P.A. CW4’s ability to dominate in the absence of AQ production.

The survival patterns of *S. aureus* in mismatched pairings also support the idea that strain adaptation influences cross-species interactions. S.A. CW2 survived only in co-culture with its original *P. aeruginosa* partner, suggesting prior adaptation to the specific competitive pressures exerted by P.A. CW2. In contrast, exposure to P.A. CW4 resulted in rapid decline. The emergence of SCVs by day 4 in S.A. CW4 further supports the idea of stress-induced adaptation. SCV formation in *S. aureus* has been linked to prolonged exposure to HQNO and other stressors and reflects a survival strategy against biofilm-associated pressures [27]. Importantly, *S. aureus* has been shown to adapt to *P. aeruginosa* through mutations in membrane transport systems involved in compound uptake and through shifts in metabolic activity that promote increased amino acid uptake [28,29]. A single mutation in *tycA*, which encodes a transmembrane transport unit in *S. aureus*, confers moderate protection against *P. aeruginosa*-produced PQS [30]. This could explain the observed S.A. CW2 survival when paired with AQ producing P.A. CW2. These adaptations, which may confer resistance to specific *P. aeruginosa* strains, appear ineffective when exposed to genetically and phenotypically distinct competitors. The ability of AQ-deficient P.A. CW4 to kill *S. aureus* suggests that AQ production is not strictly required for dominance in a dual-species biofilm. While AQs are established as antimicrobial agents, other virulence factors produced by *P. aeruginosa* such as rhamnolipids, phenazines and hydrogen cyanide also contribute to bacterial virulence [31,32]. However, AQs also regulate the production of these diverse exoproducts that inhibit *S. aureus* growth [12]. The fact that P.A. CW4 can still outcompete S.A. CW2 in the absence of AQ production highlights the potential compensatory or dominant role of alternative mechanisms which could be isolate specific. This underscores the need to investigate alternative mechanisms and cautions against extrapolating findings from laboratory strains to clinical contexts. Laboratory-adapted *P. aeruginosa* strains differ substantially from clinical isolates in their antibiotic susceptibility profiles, virulence potential and genomic content, including the presence of unique pathogenicity islands [33].

Overall, this study highlights the functional diversity of *P. aeruginosa* and the importance of clinical isolate selection in understanding polymicrobial infection dynamics. As the conclusions are drawn from a focused analysis of two matched clinical isolate pairs without the presence of non-coinfecting control strains, further work is needed to determine the extent to which these findings can be generalized. Microscopy should also be conducted on the clinical isolate pairs in the mCWB model to determine potential phenotypic/architectural differences in biofilm formation between the different strain combinations. However, the data offer a useful starting point for understanding dual-species interactions in co-isolated clinical strains. Future studies should also aim to track co-culture outcomes of mismatched clinical pairs over time to identify the genomic, transcriptomic and proteomic changes that govern survival and competition.

## Abbreviations

AQs: 2-alkyl-4-quinolones
CF: cystic fibrosis
c.f.u: colony forming units
C9-PQS: 2-nonyl-3-hydroxy-4(1*H*)-quinolone
HHQ: 2-heptyl-4-hydroxyquinoline
HQNO: 2-heptyl-4-hydroxyquinoline-*N*-oxide
LB: lysogeny broth
LC-MS/MS: liquid chromatography-MS
LT: long-term
mCWB: modified chronic wound biofilm
MSA: Mannitol salt agar
NHQ: 2-nonyl-4-hydroxyquinoline
NQNO: 2-nonyl-4-hydroxyquinoline-*N*-oxide
OM: osteomyelitis
PIA: Pseudomonas isolation agar
PQS: Pseudomonas quinolone signal
QS: quorum sensing
SCVs: small colony variants
WSM: wound simulating media
